# Association Between the Affordable Care Act and Emergency Department Visits for Psychiatric Disease

**DOI:** 10.5811/westjem.57630

**Published:** 2023-05-05

**Authors:** Afsaneh Asgharian, Jane B. Neese, M. Lori Thomas, A. Suzanne Boyd, Yvette M. Huet

**Affiliations:** College of Health and Human Services, University of North Carolina at Charlotte, Charlotte, North Carolina

## Abstract

**Introduction:**

Emergency department (ED) utilization for psychiatric disease is increasing, and a lack of health insurance has been identified as a potential cause of preventable or avoidable ED use. Through the Affordable Care Act (ACA), more uninsured individuals gained health insurance; however, the effects of increased health insurance coverage on ED utilization for psychiatric disease have not been examined.

**Methods:**

We performed a longitudinal, cross-sectional analysis of data from the Nationwide Emergency Department Sample, the largest all-payer ED database in the US, which contains data on over 25 million ED visits each year. We examined ED utilization for psychiatric disease as the primary reason for visit among adults aged 18–64. We compared the proportion of ED visits with a psychiatric diagnosis during post-ACA years (2011–2016) to pre-ACA (2009) using logistic regression adjusted for age, gender, payer, and hospital region.

**Results:**

The proportion of ED visits with psychiatric diagnosis increased from pre-ACA (4.9%) to post-ACA years (ranging from 5.0–5.5%). There was a significant difference in the proportion of ED visits with a psychiatric diagnosis when comparing each post-ACA year with pre-ACA, with adjusted odds ratios ranging from 1.01–1.09. Among ED visits with a psychiatric diagnosis, the most common age group was 26–49 years, and patients were more likely to be male than female and to have visited urban rather than rural hospitals. During post-ACA years (2014–2016), private and uninsured payers decreased, Medicaid payers increased, and Medicare payers increased in 2014 and decreased in 2015–2016 compared to pre-ACA.

**Conclusion:**

With the ACA more people gained health insurance, yet ED visits for psychiatric disease continued to increase. These results suggest that increasing access to health insurance alone is not sufficient to reduce ED utilization for patients with a psychiatric disease.

## INTRODUCTION

Mental disorders are common in the United States (US), with about 25% of adults with a mental disorder^1^,^2^ and 22.3% with serious mental disorders.^1^ However, 57.4% of adults with mental disorders and 33.3% of those with serious mental disorders did not receive mental health services in the past year.^3^ Multiple studies have shown that psychiatric disorders account for a large and growing number of all emergency department (ED) visits in the US.^2^,^4^–^7^ From 1992 to 2000, psychiatric-related ED visits for adults increased by 15%.^4^ Visits to the ED increased by 8.6% from 2006 to 2011 for all adults aged 18–64, but they increased by 20.5% for primary psychiatric diagnoses and by 53.3% for ED visits with psychiatric comorbidity.^6^

The number of ED visits for patients with psychiatric disease continues to increase. With increasing numbers of psychiatric patients and limited availability or access to outpatient, mental healthcare facilities, psychiatric patients turn to EDs for their healthcare.^8^ The Affordable Care Act (ACA) improved access, affordability, and quality of healthcare and required benefits for mental health and substance use disorder through the essential health benefits (EHB). Among the EHBs included were ambulatory and emergency services, hospitalization, and mental health and substance use disorder services, including behavioral health treatment and prescription drugs.^9^

A lack of health insurance has been identified as a contributor to the utilization of emergency services for non-emergent psychiatric conditions,^10^ and a lack of mental healthcare resources is frequently cited as a reason for seeking emergency psychiatric care.^11^,^12^ With the enactment of the ACA, the number of uninsured individuals declined by 43%, from 48.6 million in 2010 to 28.6 million in 2015,^13^ and the percentage of uninsured nonelderly adults aged 18–64 declined by 41% from 2010 to 2018.^14^ If lack of health insurance was a primary reason for ED utilization for psychiatric disease, then an increase in health insurance coverage would be expected to reduce ED utilization for patients with psychiatric diagnosis. However, whether a reduction in the uninsured population in the US is correlated to changes in ED utilization for psychiatric disease is currently unknown.

The 2016 National Hospital Ambulatory Medical Care Survey (NHAMCS) showed that nearly 5.5 million (3.8%) ED visits had a primary diagnosis of mental disorder,^7^, and the proportion of ED visits with a psychiatric diagnosis increased from 4.1% to 5.4% between 2007– and 2016.^15^ However, that survey did not specifically examine the effect of the ACA on ED visits with psychiatric diagnosis from post-ACA compared to pre-ACA years. In our study, we used nationally representative ED discharge data from pre-ACA and post-ACA years to compare the proportion of ED visits for psychiatric disease in years before versus after implementation of the ACA. Our primary objective in this study was to evaluate the association between the ACA and ED visits for patients with psychiatric disease for post-ACA years 2011–2016 and pre-ACA year 2009.

## METHODS

### Standard Protocol Approvals, Registrations, and Patient Consents

This study was exempt from approval by the University of North Carolina at Charlotte Institutional Review Board due to the fully de-identified nature of the data, and informed consent was not required. The analysis was compliant with the Healthcare Cost and Utilization Project data use agreement policy.

**Population Health Research Capsule**

What do we already know about this issue?

*A lack of mental health care resources is a contributor to ED use. The Affordable Care Act (ACA) improved benefits for mental health, yet ED use continues to increase*.

What was the research question?

*We evaluated the association of the ACA and ED visits for adults with psychiatric disease post-ACA, 2011–2016, and pre-ACA, 2009*.

What was the major finding of the study?

*The analysis of ED visits with primary psychiatric diagnosis for post-ACA 2016 vs pre-ACA year was significant with odds ratio (95% CI) of 1.040 (1.037, 1.043)*.

How does this improve population health?

*Increasing access to health insurance alone is not sufficient to reduce ED use by patients with psychiatric disease*.

### Data

We used data from the Nationwide Emergency Department Sample (NEDS), which is the largest all-payer ED database in the US. The NEDS consists of all ED visits occurring at one of over 950 hospital-based EDs with more than 25 million unweighted observations per year.^16^ The NEDS database consists of ED visit-level discharge data. Diagnoses or disease conditions were coded and collected for each ED visit with up to 15 diagnoses for 2009–2013 and up to 30 diagnoses for 2014–2016. According to ambulatory coding guidelines, the diagnosis code in the first position indicates the primary reason for the healthcare encounter and is thus referred to as the “primary diagnosis”; all other diagnoses are placed in a secondary position. The diagnosis codes of NEDS were based on the *International Classification of Diseases*, 9^th^ Clinical Modification (ICD-9-CM) for the years 2009–2014 and the first three-quarters of 2015 and the 10^th^ Clinical Modification (ICD-10-CM) for the last quarter of 2015 and 2016. We used data from 2009 and 2011–2016 for analysis.

### Population

We identified all ED visits for adults aged 18–64 with psychiatric diagnoses in the primary diagnosis position (ICD-9-CM 290-319 and ICD-10-CM F01-F99). We excluded adults ≥65 years because Medicare is the primary insurer for older adults and was not affected or designed to be impacted by the ACA. We categorized psychiatric diagnoses by ICD-9-CM code into the following categories: Dementias/Delusional/Transient/Persistent (290, 293, 294, 297); Drug and Alcohol Dependence (291, 292, 303, 304, 305); Schizophrenic and Other Psychoses (295, 298); Depressive and Episodic Mood (296, 311); Anxiety, dissociative and somatoform (300); Acute and Adjustment Reaction to Stress (308, 309); and Other (299, 301, 302, 306, 307, 310, 312–319). Since the last quarter of 2015 and the 2016 data had ICD-10 codes, all ICD-9 codes were mapped to ICD-10 detailed levels using the Agency for Healthcare Research and Quality MAPIT toolkit. The MAPIT tool takes a set of ICD-9 codes at each level, up to five digits, and maps them to equivalent ICD-10 codes at each level up to five digits using the Centers for Medicare & Medicaid Services equivalence mapping.^17^

### Outcomes and Covariates

The primary endpoint was the proportion of ED visits with a psychiatric diagnosis in the primary diagnosis position. This endpoint was summarized for each pre-ACA and post-ACA year, and each post-ACA year was compared with the pre-ACA reference using logistic regression adjusting for the following covariates: age (18–25, 26–49, 50–64); gender; payer (Medicare, Medicaid, private, uninsured); and hospital region (urban, rural). Race is not recorded in the NEDS database. We performed adjusted and unadjusted analyses of the proportion of ED visits with primary psychiatric diagnosis for each post-ACA year compared to pre-ACA. For statistical analyses, all tests were two-sided, with significance interpreted at α = 0.05. The odds ratio (OR) and 95% confidence interval (CI) using Mantel-Haenszel chi-Square (χ^2^) statistics were reported. We used SAS statistical software version 9.4 (SAS Institute, Inc, Cary, NC) for all analyses. The study results are reported using the Strengthening the Reporting of Observational studies in Epidemiology (STROBE) guidelines.^18^

## RESULTS

The number of observed annual ED visits for adults aged 18–64 was 17.6 million in 2009 and ranged from 17.8 million to 20.1 million for 2011–2016 (Table 1). The number of ED visits with primary psychiatric diagnosis was more than 866,000 in pre-ACA 2009 and increased to between 892,000 and 1.1 million in post-ACA years. The proportion of ED visits with primary psychiatric diagnosis for pre-ACA and post-ACA years increased from 4.9% in 2009 to ranging from 5.0% to 5.2% in 2011–2013, and between 5.3% to 5.5% in 2014–2016 (Table 1). The number of ED visits continued to increase in each post-ACA year compared to pre-ACA, with more than one million ED visits with primary psychiatric diagnosis each year from 2014–2016. In both adjusted and unadjusted analyses, there was a statistically significant difference in the proportion of ED visits with primary psychiatric diagnosis for each post-ACA year compared to pre-ACA 2009 with adjusted ORs ranging from 1.01–1.09 and unadjusted ORs ranging from 1.02 to 1.12 (Table 2). All adjusted and unadjusted analyses of the proportion of ED visits with psychiatric diagnosis and all covariates (age, gender, payer, hospital region) were statistically significant (P<0.001).

The most common age group among ED visits with primary psychiatric diagnosis was 26–49 (54.2% to 57.5%) followed by 50–64 (22.6% to 26.1%), and 18–25 (19.5% to 20.6%). Males (55.3% to 57.9%) had more ED visits with primary psychiatric diagnosis than females (42.1% to 44.7%) (Table 3).

The proportion of ED visits with primary psychiatric diagnosis where Medicare was the primary expected payer increased from 14.5% in 2009 to 15.2% in 2014 and decreased to 14.2% in 2015 and 14.0% in 2016. For Medicaid, it increased from 25.7% in pre-ACA 2009 to 28.7% in 2013 and continued to increase in post-ACA 2014–2016 from 36.1% to 38.8%. There was a decrease in the proportion of ED visits with primary psychiatric diagnosis for the private payers from 25.9% in 2009 to 22.4% in 2013, and between 22.7% to 24.3% in 2014–2016. The same trend was observed for the uninsured payers with the proportion of ED visits of 33.8% in 2009, 32.2% to 33.7% in 2011–2013 with significant decrease from 26.1% in 2014 to 23.2% in 2016 (Table 4). There was a significant increase in the proportion of ED visits for Medicaid payers and a significant decrease for uninsured payers in post-ACA 2014–2016 compared to pre-ACA and the other post-ACA years.

The proportion of ED visits with primary psychiatric diagnosis for urban hospitals increased from 95.6% in pre-ACA 2009 to post-ACA 2011 to 2015, ranging from 95.8% to 97.1%, and decreased to 95.7% in 2016. For rural hospitals, the proportion of ED visits decreased from 4.4% in pre-ACA 2009 to 2.9% in 2015 and 4.3% in 2016 (Table 4).

The most common primary psychiatric diagnoses were for Drug/Alcohol disorders (34.8% to 43.1%), followed by Depressive (19.4% to 25.8%) and Anxiety disorders (17.9% to 19.4%), and other psychiatric diagnoses, Schizophrenic disorders (11.3% to 13.5%), Dementia (0.7% to 1.0%), and Stress (3.0% to 3.6%) (Table 5).

## DISCUSSION

The proportion of ED visits and ED visits with psychiatric diagnosis for adults aged 18–64 increased in the post-ACA years compared to pre-ACA. Even when accounting for the increase in ED volume over time, the proportion of ED visits with psychiatric diagnosis increased and was higher in post-ACA years 2014–2016 than pre-ACA year and other post-ACA years. These results suggest that increasing access to health insurance alone is not sufficient to reduce ED utilization for patients with a psychiatric disease.

We found that psychiatric diagnoses accounted for 4.9% in 2009 and 5.0–5.5% of ED visits in the US from 2011–2016. This is consistent with findings from the NHAMCS, where the proportion of ED visits with a psychiatric diagnosis for adults ≥19 years was 4.3–5.4% from 2009–2016.^15^

With the ACA, more people gained health insurance, and insurance plans were required to include mental health and substance use disorder treatment and services as part of the EHBs.^19^ Yet the number and proportion of all ED visits and ED visits for patients with psychiatric diseases continued to increase and was significantly higher for post-ACA than pre-ACA years in our study. A lack of health insurance limits access to outpatient mental health services. If this was the primary driver of ED utilization for psychiatric disease, then increased insurance coverage should have led to greater outpatient mental health services utilization, which would in turn have decreased ED utilization for psychiatric disease. However, we observed the opposite trend: ED visits for psychiatric disease increased despite greater insurance coverage.

There are several potential reasons for the paradoxical increase in ED visits for psychiatric disease despite greater insurance coverage. First, health insurance coverage is not a guarantee of outpatient healthcare access. For example, people with health insurance may not seek medical or psychiatric care due to access barriers, lack of a designated primary care or mental health physician, or high out-of-pocket costs. In addition, some mental health clinicians may not accept certain health insurance, such as Medicaid. Second, having mental healthcare access alone may not sufficiently prevent psychiatric emergencies from occurring. Although psychiatric diseases can be managed in an outpatient setting, some conditions may require emergency care, and not all ED visits for psychiatric diseases are preventable or avoidable.^2^,^20^

Psychiatric-related ED visits have continued to increase, both before and after the ACA. Much of the literature on preventable ED visits for psychiatric disease has focused on a lack of alternative healthcare resources for acute psychiatric disease. With the replacement of state mental hospitals by community mental health centers, there has been a shift from inpatient to outpatient care, which has left some patients with severe mental illness at risk for lapses in care and readmission. Furthermore, community mental health centers have declined over time due to a lack of state and local funding. As a result, in 2003 the Subcommittee on Acute Care to the President’s New Freedom Commission reported that since 1970, the total number of inpatient psychiatric beds per capita and the number of state and county psychiatric beds per capita declined by 62% and 89%, respectively.^21^

We observed that the most common psychiatric diagnosis was Drug and Alcohol disorders followed by Depressive, Anxiety and Schizophrenic disorders. We also observed a change in the payer mix for ED visits with primary psychiatric diagnosis before vs after passage of the ACA. The ACA aimed to increase access to health insurance through two different mechanisms: 1) enhanced access to private health insurance plans through the marketplace; and 2) an increase in Medicaid eligibility.^19^ In our study, a decrease in uninsured patients was offset by an increase in Medicaid-insured patients but not by private payers. This suggests that the effect of the ACA on uninsured ED visits with primary psychiatric diagnosis was mediated by Medicaid rather than private insurance.

## LIMITATIONS

Our study had several limitations. First, while the ACA was passed in 2010, it was not fully implemented until January 1, 2014. Therefore, the classification of 2011–2013 as post-ACA years may have been incomplete. We were also limited to a single pre-ACA year. The sampling strategy of NEDS includes government, non-federal (public), private not-for-profit, and private investor hospitals but not federal hospitals such as Veteran’s Administration, Department of Defense, and Indian Health Service hospitals, which limits generalizability. The NEDS lacks data such as severity of mental illness and outpatient treatment history. While we were able to characterize changes in payer mix among ED patients with and without psychiatric disease, we lacked data on the prevalence and insurance status of patients who were not seen in the ED. Because of the logistical barriers involved in signing up for expanded access to Medicaid benefits, patients with serious mental illness may be less likely to benefit from ACA-related coverage and are also more likely to require ED care. Furthermore, depending on the prevalence of psychiatric disease in the general population, an increasing number of ED visits could theoretically represent a relative decrease in ED utilization if the overall prevalence were increasing more rapidly.

Future studies should re-evaluate the association between the ACA and psychiatric-related ED visits as more data becomes available and possibly use a national claims database with detailed information for the ED visits. Furthermore, because Medicaid expansion varies by state, future studies should also examine state-level variability in the association between the ACA and ED visits with psychiatric diagnosis.

## CONCLUSION

We showed that with the Affordable Care Act, more people gained health insurance. However, we also showed that psychiatric-related visits to the ED increased both before and after the ACA. Even with health insurance, there are barriers to accessing outpatient mental healthcare, and even with outpatient treatment, some psychiatric emergencies are unavoidable. With the ACA, more people gained health insurance, yet ED visits for patients with primary psychiatric diagnosis continued to increase. These results suggest that increasing access to health insurance alone is not sufficient to reduce ED utilization for patients with a psychiatric disease.

## Figures and Tables

**Table 1:** Summary of emergency department visits for adults aged 18–64.

	Year	All ED visits adults 18–64 n	Primary or secondary psychiatric diagnosis adults 18–64 n (%)	Primary psychiatric diagnosis adults 18–64 n (%)
Pre-ACA	2009	17,645,539	4,253,110 (24.1)	866,810 (4.9)
	2011	17,845,772	4,717,856 (26.4)	892,511 (5.0)
	2012	19,325,068	5,267,941 (27.3)	984,964 (5.1)
Post-ACA	2013	18,412,805	5,261,875 (28.6)	963,247 (5.2)
	2014	19,498,007	5,832,444 (29.9)	1,066,007 (5.5)
	2015	18,738,803	5,857,686 (31.3)	1,018,056 (5.4)
	2016	20,073,238	5,986,274 (29.8)	1,054,731 (5.3)

Percentages are based on total visits, N.

*ACA*, Affordable Care Act; *ED*, emergency department.

**Table 2:** Analysis of emergency department visits with primary psychiatric diagnosis, post- vs pre-Affordable Care Act.

Post-ACA vs Pre-ACA	Analysis of ED visits with primary psychiatric diagnosis	

	Adjusted analysis a	Unadjusted analysis
	Odds ratio (95% CI) b	Odds ratio (95% CI) b
2011 vs 2009	1.012 (1.008, 1.015)	1.019 (1.016, 1.022)
2012 vs 2009	1.031 (1.028, 1.034)	1.040 (1.036, 1.043)
2013 vs 2009	1.060 (1.057, 1.063)	1.069 (1.065, 1.072)
2014 vs 2009	1.086 (1.083, 1.089)	1.119 (1.116, 1.123)
2015 vs 2009	1.067 (1.064, 1.071)	1.112 (1.109, 1.115)
2016 vs 2009	1.040 (1.037, 1.043)	1.073 (1.070, 1.077)

a Adjusted analysis model adjusted for age, gender, payer and hospital region.

b P-value using χ^2^ test. All statistical tests and covariates were significant (P<0.001).

*ACA*, Affordable Care Act; *ED*, emergency department; *CI*, confidence interval.

**Table 3:** Emergency department visits with primary psychiatric diagnosis by age and gender.

	Year	N	Age (Year)			Gender	
	
			18 – 25	26 – 49	50 – 64	Male	Female
Pre-ACA	2009	866,810	172,429 (19.9)	498,220 (57.5)	196,161 (22.6)	478,809 (55.3)	387,334 (44.7)
	2011	892,511	183,478 (20.6)	493,806 (55.3)	215,227 (24.1)	498,973 (55.9)	393,473 (44.1)
	2012	984,964	199,197 (20.2)	538,987 (54.7)	246,780 (25.1)	549,789 (55.8)	435,123 (44.2)
Post-ACA	2013	963,247	193,247 (20.1)	524,007 (54.4)	245,993 (25.5)	543,271 (56.4)	419,915 (43.6)
	2014	1,066,007	210,498 (19.8)	577,819 (54.2)	277,690 (26.1)	612,818 (57.5)	453,152 (42.5)
	2015	1,018,056	198,452 (19.5)	555,257 (54.5)	264,347 (26.0)	589,326 (57.9)	428,642 (42.1)
	2016	1,054,731	205,351 (19.5)	578,560 (54.8)	270,820 (25.7)	610,655 (57.9)	443,966 (42.1)

Percentages are based on total visits, N.

*ACA*, Affordable Care Act; *ED*, emergency department.

**Table 4:** Emergency department visits with primary psychiatric diagnosis by payer and by hospital region.

	Year	N	Payer				Hospital region	
	
			Medicare	Medicaid	Private	Uninsured	Urban	Rural
Pre-ACA	2009	866,810	125,094 (14.5)	221,664 (25.7)	223,359 (25.9)	290,938 (33.8)	828,261 (95.6)	38,549 (4.4)
	2011	892,511	134,890 (15.2)	262,006 (29.5)	205,794 (23.2)	286,005 (32.2)	855,365 (95.8)	37,146 (4.2)
	2012	984,964	149,962 (15.3)	286,841 (29.2)	219,056 (22.3)	327,253 (33.3)	949,855 (96.4)	35,109 (3.6)
Post-ACA	2013	963,247	146,099 (15.2)	276,061 (28.7)	215,432 (22.4)	323,488 (33.7)	927,678 (96.3)	35,569 (3.7)
	2014	1,066,007	161,764 (15.2)	383,703 (36.1)	241,292 (22.7)	277,494 (26.1)	1,027,502 (96.4)	38,505 (3.6)
	2015	1,018,056	144,011 (14.2)	393,715 (38.8)	237,632 (23.4)	240,764 (23.7)	988,728 (97.1)	29,328 (2.9)
	2016	1,054,731	146,990 (14.0)	405,756 (38.5)	256,005 (24.3)	244,169 (23.2)	1,009,363 (95.7)	45,368 (4.3)

Percentages are based on total visits, N.

*ACA*, Affordable Care Act; *ED*, emergency department.

**Table 5:** Summary of primary psychiatric diagnoses.

Year	N	Dementia	Drug / alcohol	Schizophrenic	Depressive	Anxiety	Stress	Other
2009	866,810	5,887 (0.7)	301,495 (34.8)	110,006 (12.7)	223,398 (25.8)	155,117 (17.90)	28,340 (3.3)	42,567 (4.9)
2011	892,511	5,982 (0.7)	331,756 (37.2)	106,734 (12.0)	207,067 (23.2)	165,474 (18.5)	31,968 (3.6)	43,530 (4.9)
2012	984,964	6,586 (0.7)	359,446 (36.5)	121,017 (12.3)	234,250 (23.8)	184,013 (18.7)	32,785 (3.3)	46,867 (4.8)
2013	963,247	6,900 (0.7)	357,571 (37.1)	119,226 (12.4)	224,840 (23.3)	175,901 (18.3)	32,716 (3.4)	46,093 (4.8)
2014	1,066,007	7,804 (0.7)	393,245 (36.9)	144,214 (13.5)	245,995 (23.1)	192,138 (18.0)	32,891 (3.1)	49,720 (4.7)
2015	1,018,056	8,577 (0.8)	420,313 (41.3)	117,213 (11.5)	208,329 (20.5)	188,095 (18.5)	30,677 (3.0)	44,852 (4.4)
2016	1,054,731	10,498 (1.0)	454,532 (43.1)	118,775 (11.3)	204,245 (19.4)	204,684 (19.4)	33,482 (3.2)	28,515 (2.7)

Percentages are based on all psychiatric diagnoses, N.
